# Trauma-Informed Caregiving with Older Adults: Applying a Conceptual Framework Using an Existential Lens for Practice

**DOI:** 10.3390/healthcare14060740

**Published:** 2026-03-14

**Authors:** Rebecca L. Koltz, Daniel J. Koltz

**Affiliations:** 1 Department of Counseling, Montana State University, Bozeman, MT 59717, USA; 2 Department of Human Development & Community Health, Montana State University, Bozeman, MT 59717, USA; daniel.koltz@montana.edu

**Keywords:** trauma, older adults, caregivers, EITC framework, existential caregiving

## Abstract

In the wake of trauma, individuals often experience not only psychological distress but also a profound disruption in their sense of self, meaning, and existence. This paper explores trauma-informed caregiving through an existential lens, emphasizing the need to address both the emotional and philosophical dimensions of trauma. While traditional trauma-informed approaches prioritize safety, trust, and empowerment, an existential perspective expands this foundation by acknowledging humans’ struggle with freedom, isolation, meaninglessness, and mortality. Utilizing an existential conceptual framework that incorporates trauma-informed care to improve person-centered practices is particularly applicable to caregiving with older adults. Encouraging caregivers to cultivate authentic presence, foster autonomy, and support the individual’s capacity to reframe their current life experience. By incorporating psychological insight with existential depth, this approach promotes the reawakening of purpose, agency, and connection while understanding the impact that trauma has in the caregiving process for both the caregiver and the person needing care. Case studies, practical strategies, and emerging research are discussed to support the implementation of this compassionate and holistic caregiving paradigm.

## 1. Introduction

Trauma is a word that has received significant attention in recent years. Much of the writing and research on trauma in caregiving has centered on parents and their caregiving roles [1]. However, far less attention has been given to the experience of caregiving for older adults, leaving an important gap in both literature and practice. Trauma is not a concept that escapes certain times in the lifespan, and one might hypothesize that at end stages of life, it may surface with renewed intensity as individuals face loss, vulnerability and the inevitable complexity of a declining state of health. While caregiving of older adults does not always involve dementia, Havermans et al. [1] emphasized that little information is known about the prevalence of post-traumatic stress disorder, particularly among individuals with dementia. This gap highlights the limited understanding of older adults’ experiences and the ways in which trauma may resurface during this stage of life for both the caregiver and the person needing care. Havermans et al. [1] further state that most nursing staff receive insufficient training regarding the impact of trauma. The authors will apply a new conceptual Existential Trauma-Iinformed care (ETIC) framework that utilizes common approaches to improve person-centered practices. This framework uses the four primary concepts of existentialism and creates an understanding for how to use trauma-informed practices within those constructs to improve care [2], creating a more comprehensive understanding of how to care for older adults and understand the trauma they are experiencing as they transition to their individual situations [2]. ETIC was developed to help caregivers compassionately and relationally address potential trauma that gets in the way of some care that older adults need but are unwilling to engage in.

## 2. Types of Caregiving

Caregiving is most generalized as two different types, either family or professional care [3]. Family caregivers are the most recognized caregivers in research, as the largest share of care is provided through this method. According to the National Alliance for Caregiving [3], there are over 63 million family caregivers providing some level of care for a person in need of support for activities of daily living (ADLs) or instrumental activities for daily living (IADLs). Some family caregivers may not be relatives; friends, neighbors, and other volunteers can fill the gaps in care with minimal caregiving activities.

The second type of caregiving is care provided by professional caregivers. According to Scales and Mcall [4] from the Paraprofessional Healthcare Institute, there are over 5.4 million direct-care professional caregivers, comprising in-home care workers or other professionals employed by various types of facilities that house older adults in need of assisted care. Often, bathing, skilled nursing, medication management, and health monitoring are the duties performed as part of professional caregiving tasks. These facilities provide housing in independent living, assisted living, skilled nursing, or medical settings. Professional caregivers, whether they provide home care or care in a facility, have access to training and guidance on best practices for care [4].

The three levels of care provided by caregivers are informal care, intermediate care, and professional caregiving [5]. Informal care is provided for older adults who need basic assistance to aid in their daily living routines. This type of care is most often provided by the family caregiving system. Intermediate care is often needed for more instrumental activities that include healthcare management of chronic illness, disease, or disability [5]. The person needing care cannot function without these necessary caregivers at this level. Both family caregivers and professional caregivers may be utilized during this stage of care. The third level of care is when professional care is required to fill any gaps in care, starting with the first level if a family caregiver is not available; however, most often this level is when it is medically necessary for the safety and health of the individual. Older adults at this stage may go through a variety of emotional states, as it is often the result of a traumatic health event that leads to the realization that their life is now forever changed [5].

Professional caregivers are in a position of influence when someone needs their services. The professional caregiver has access to training and could be trained to engage in the existential experience an individual may be going through as part of a person-centered approach as they encounter these life changes [5]. Therefore, trauma-informed caregiving education is best targeted at professional caregivers.

## 3. Trauma-Informed Caregiving

Trauma-informed caregiving (TIC) for older adults involves recognizing the impact of trauma on individuals and providing care that is sensitive and responsive to their needs. By older adulthood, individuals have typically experienced at least one traumatic event (50–90%) [6]. Not all these individuals develop post-traumatic stress disorder (PTSD); however, even if there was no initial PTSD diagnosis, trauma can re-emerge later in the lifespan as individuals encounter the typical experiences of aging (illness, loss, retirement, decreased mobility, etc.). Trauma-informed caregiving recognizes that symptoms of early traumatic experiences may manifest later in life and is guided by core principles, including recognizing trauma, establishing safety, fostering trustworthiness, facilitating peer support, promoting collaboration, empowering individuals, and addressing cultural, historical, and gender-related factors [7]. Currently, there are no trauma-informed practices specifically for long-term care facilities [6]; therefore, the authors of this article are using SAHMSA [7] evidence-informed principles noted above in conjunction with existential concepts. Research acknowledges that PTSD is prevalent in older adult populations [7], yet no formal training exists to provide trauma-informed care for these individuals. Caregivers can support older adults who have experienced trauma with some basic practices, thus creating safe and supportive environments through patience, kindness, and understanding while validating feelings within clear and respectful boundaries.

Some essentials of trauma-informed care include being mindful of language and personal self-care as a caregiver [8]. Using trauma-informed language is essential to avoid blame or judgment and instead focus on empowerment. For example, rather than asking, “What is wrong with you?” which may be triggering, a caregiver could say, “How can I support you right now?” Being mindful of language and its potential impact on the older adult is critical. Effective caregiving also requires self-care, ensuring caregivers maintain their own well-being so they can provide consistent, compassionate support.

Because trauma affects each person differently, interventions should be tailored to individual needs and stage of life [2]. For instance, a seemingly ordinary conversation about expressing love or saying goodbye may be profoundly difficult for someone who has experienced abuse or unaddressed childhood trauma. Finally, caregivers can foster empowerment by involving older adults in decision-making, offering choices, and recognizing their strengths. These practices not only support autonomy but also contribute to a greater sense of dignity and self-worth, often aligning well with person-centered care.

While person-centered care has been the gold-standard framework for aging people, there are critiques of this framework that Existential Trauma-Informed care (ETIC) addresses. The Administration for Community Living [9] uses the term person-centered, trauma-informed (PCTI), integrating the principles of person-centered care with trauma-informed approaches [8]. Each is from a humanistic philosophical perspective; however, existentialism leans into many of the realities of care and could be combined to create a person-centered approach to care. In lieu of trauma-informed models specifically for care of older adults, person-centered care is the standard of care. In the delivery of a person-centered approach for care, each care plan is tailored to match the wishes of the individual [10]. The lack of structure, however, neglects the basic needs of older adults to function healthily, and their needs remain unmet. In many situations, the older adult has self-agency over what they do and do not do daily. These approaches are not always suitable for more extreme conditions, such as emotional distress, social isolation, and mental health issues related to their worsening health condition [11]. For example, engaging in a basic activity in the care plan, such as going to a social function they were excited to attend earlier, raises the question: if they decide against it, where is the line drawn between fostering social isolation and respecting the wishes of the individual in the original care plan? The caregiver should question the individual by asking them why they do not want to engage in a social activity today, rather than saying, “Ok, we would not take you to the activity.”

The existential approach focuses on asking questions in a way that encourages reflection, thought and consideration of a person’s overall health and on engaging in their current journey and developing a care plan together that benefits their emotional well-being. The individual needing care could benefit from exploring or understanding the trauma they have experienced to improve their outlook on their current circumstances. Improving health and well-being outcomes is as vital as the status of their person-centered care plan. When a caregiver works at the existential level with an older adult, they can engage in understanding the deeper meaning of their relationship and uncover a path to a healthier, person-centered care approach [11].

## 4. Existential Trauma-Informed Caregiving (ETIC)

While the above offer some traditional ways that trauma is addressed in caregiving, training on TIC is lacking in most medical facilities, especially for older adults. In large part because there are no training models available that adequately address how caregivers for older adults should be trained [6]. An existential philosophy may provide some additional insights and understanding of the experience of older adults. Existential philosophers and theorists posit that the search for meaning is a universal endeavor [12], and trauma significantly disrupts the meaning-making process [13]. Developed by the authors, an Existential Trauma-Informed Caregiving (ETIC) framework lends itself to a deeper understanding of the impact of trauma. Existential frameworks cultivate authentic presence, foster autonomy, and support the individual’s capacity to reframe their current life experience.

The ETIC will not only reduce harm or distress but also empower meaning-making, restore agency, and foster authentic presence, enabling individuals to reframe their life narratives in ways that promote healing, dignity, and growth. Meaning-based theories and practices such as existentialism have held that trauma impedes the meaning-making process; therefore, the application of the four existential givens of freedom, meaning, isolation, and death [14] to a trauma-informed approach to caregiving with older adults offers a framework to support care practice. At later stages of life, older people in care struggle with making meaning of their life in the caregiving experience; ETIC recommends meaning-based approaches to care integrated with SAMHSA’s [7] principles for trauma-informed caregiving with older adults.

Yalom [14] parses existential concerns into four givens: death, freedom, isolation and meaning. Yalom and other existential theorists proposed that confronting death, its inevitability and the distress that it creates, is a key issue for truly living [15]. Furthermore, avoidance of death anxiety is typical and often intensifies psychological distress. Existential concepts of death have also been applied to nonphysical losses, such as the death of a career [15]; therefore, in the context of caregiving, it is reasonable to extend this concept to the loss of anticipated retirement plans, a healthy existence in later life, and the physical capability to be active.

Another given is freedom, which is a reaction to determinism [15]. Individuals have free will and the capacity to make choices in their life; however, caregivers can help them understand factors informing their choices and support them in making alternative choices. Freedom at the heart is about agency and an individual’s ability to find agency in their experience. Yalom [14] defined isolation as a separation or chasm between the individual and the world. Certainly, for older adults receiving care, there is often a physical chasm between them and the world. However, isolation also references an intrapersonal experience [16]. Everyone is responsible for their life; one is alone and must encounter that. There are elements of the caregiving experience, especially the last stages, that a caregiver will not be able to understand. The last existential concept is meaning. Schnipke and Mackay [15] noted that the existential concept of meaning places a lot of responsibility on the individual to find their meaning. Meaning and lack of purpose may fluctuate for care receivers, especially during times of significant change, such as circumstances resulting in the caregiving experience. It can be difficult to hang on to threads of meaning when life circumstances change in unexpected ways. Yet, exploring and sustaining meaning in all circumstances remains essential at this stage of life.

### 4.1. Existential Trauma-Informed Caregiving (ETIC) Framework

**Figure 1:**
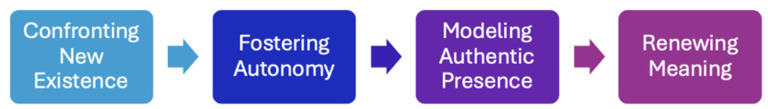
ETIC framework.

According to the CDC [17], trauma-informed approaches are less about single techniques; rather, trauma-informed care is an attitude of awareness and sensitivity and may require change at individual as well as organizational levels. The author’s Existential Trauma-Informed Caregiving (ETIC) Framework is an approach that uses current practices but integrates the existential concepts of death, freedom, isolation and meaning described above into trauma-informed standards of care outlined by SAMHSA [7]. With personal and organizational awareness and sensitivity in mind, the ETIC framework consists of four components integrating an existential perspective with trauma-informed caregiving: 1. confronting new existence, 2. fostering autonomy, 3. modeling authentic presence, and 4. renewing connection. Each component is described below in greater detail with application to formal caregiving contexts (see Figure 1).

### 4.2. Confronting New Existence: Preparing for Safety

For the CDC [17] and SAMHSA [7], the trauma-informed approach notes safety first. This means ensuring that both residents and staff feel physically and psychologically safe. When exploring this concept through an existential lens, caregivers create space for what is known as ontological security. What is ontological security? It is about creating space for a felt sense that one’s existence is stable, acknowledged, and held, while living within physical or emotional limitations [18]. Existentialism acknowledges that confronting death, existence, and the various meanings these hold for an individual is central to accepting existential reality. For older adults receiving caregiving in residential settings or at home by paid caregivers, this is recognizing that individuals in your care may be emotionally dealing with grief related to their current existence or changes in their life experience. Statements such as “cheer up” or “you are much better off” may not be helpful. Instead, an existential trauma-informed approach acknowledges trauma and seeks to understand the person’s current experience. Caregivers can support older adults by helping them reflect on their current situation and what contributes to their sense of health and safety. For example, a caregiver might say, “I understand that you did not expect to be here and that you feel sad. I wonder what could help, even a little bit, for you to feel better here?” Reflective questions, rather than statements, help care receivers process their experiences instead of repressing them, which can later emerge as depression, verbal aggression, or in some cases, physical aggression. A sense of safety is grounded in opportunities to reflect on acceptance of one’s life stage, unexpected transitions from original retirement plans, and to receive relational attunement from caregivers.

### 4.3. Fostering Autonomy: Facilitating Choice, Voice, and Empowerment

From an existential perspective, fostering autonomy addresses the existential given of freedom, specifically the freedom that exists within lived experience. Fostering autonomy aligns well with the principles from trauma-informed care—empowerment, voice and choice [5]. However, existential freedom expands these concepts by encouraging personal responsibility to define the self, rather than blaming people, situations or circumstances. As noted earlier in the article, some person-centered care approaches do not sufficiently attend to the lack of structure and basic needs that enable older adults to function with meaning and purpose. From the ETIC framework, caregivers seek to understand the deeper meaning around care receivers’ choices, rather than simply accepting them when those choices may be counterintuitive to creating meaning in their caregiving context. While choice and empowerment remain central, an applied existential framework invites deeper reflection about choices that may not support the meaning an older adult can still create within their present circumstances. In this way, ETIC encourages caregivers to facilitate reflection around values-based choices that align with care receivers’ evolving identity in the context of change. It invites the question, “Does this choice reflect the identity I am striving to create even in this circumstance?” Perhaps, a person in one’s care chooses not to get dressed; a person-centered approach may accept this decision without further exploration. In contrast, the ETIC framework would encourage reflection on whether this choice aligns with the meaning the care receiver is attempting to create in their new lived experience, thereby creating a gentler challenge. This approach invites choices of engagement, rather than passivity, in daily routines and activities, which could enhance well-being.

### 4.4. Modeling Authentic Presence: Fostering Trustworthiness and Transparency

For the ETIC framework, caregivers who model authentic presence help combat the isolation that older adults often experience. Trauma-informed models [7] discuss fostering trustworthiness and transparency but do not address the relationship that envelops that experience. From an ETIC perspective, caregivers invite authentic, human-to-human presence to model authenticity and create a caregiving experience that is a dialog rather than only a service.

Why is this important? Havermans et al. [1] noted that residential treatment staff receive limited education on the effects of trauma. In general, nursing staff receive little education on trauma or psychological literacy and call for more trauma-informed approaches, specifically in nursing care settings. However, engaging in trauma-informed care involves forming a relationship with the care receiver, humanizing them, and not simply providing a service. To model authentic presence means engagement with oneself and being aware of your own reactions, let alone the reactions of the person in your care [19]. Fostering self-awareness and authentic presence allows you to care so that you can be present for others and foster an environment that promotes healing. The reflective practice begins within: “How am I feeling in this moment? How present am I?” This type of reflection makes room for caregivers to be more balanced in stressful situations and moments, responding rather than reacting.

Modeling authentic presence calls for cultivating self-awareness, not just procedural clarity in the caregiving process. For example, it may mean taking care of the tasks associated with caregiving and taking a moment to authentically connect when the individual seems upset, depressed, or anxious. To do this, the caregiver must be present, not thinking about the task list ahead. With this we are not assuming that schedules are not important; rather, we emphasize being aware of the extra moments that caregivers can take with the person in their care to create a caregiving experience that addresses isolation and fosters greater connection [19].

### 4.5. Renewing Meaning: Facilitating Collaboration and Mutuality

The final component of ETIC is renewing meaning. To review, the previous components include confronting new existence through reflective questions, fostering autonomy, modeling authentic presence, and now renewing meaning. Renewing meaning builds upon these foundations through the integration of collaboration and mutuality, core principles of trauma-informed care outlined by [7].

Embedding collaboration and mutuality within an existential framework creates opportunities for renewed meaning and for an older adult’s connection with meaning. While collaboration and mutuality aim to level power differences between staff and the individual, the ETIC framework understands this leveling as occurring through shared meaning-making rather than through role equalization alone. As previously stated, the caregiving relationship becomes a co-journey of meaning, not a one-sided support structure. Mutuality honors the caregiver and the individual as co-creators in the healing process. Reconnection with meaning within the caregiving experience becomes a pathway to reclaiming one’s place in the world following the existential questioning introduced in the first component of the ETIC framework. From a pragmatic perspective, this final component invites caregivers to actively encourage an older adult to reflect on purpose and meaning. While the person’s new normal is different, unexpected, or even unwelcome, continued dialog about meaning can help bridge acceptance of the new reality with the discovery of potential within that reality.

This type of reflection requires the caregiver to remain attentive to moments when signs of meaning-making emerge. For example, “George, I noticed that you talked about helping your friend today. You have not talked about that for a long time. You sound happy.” On days when George appears discouraged, the caregiver might gently remind him that helping his friend made a meaningful difference in that person’s life. A caregiver may also authentically reflect on how George contributed to their own experience, such as “George, you help me as much as I help you. You smile at me every day and ask how my kids are doing.” While these may seem like small reflections, they hold significant potential to support the creation of new meaning. For a caregiver, this opens a door into the individual’s world that may not have occurred without engaging in true meaning-making and allows for deeper engagement.

To summarize, the ETIC framework uses the current trauma-informed caregiving principles laid out by SAMHSA [7] and considers them in conjunction with existential theory, thus creating a new framework to guide the caregiving process. The ETIC framework includes: 1. confronting new existence, 2. fostering autonomy, 3. modeling authentic presence, and 4. renewing meaning-making. These four areas align with the existential concepts of death, freedom, isolation and meaning. This framework calls for caregivers to become reflective caregivers not only on the experience of the care receiver, but also their own experience. With the ETIC framework, caregivers must be able to ask some of these questions of themselves.

## 5. Implementation of ETIC Framework: A Case Study

**Figure 2:**
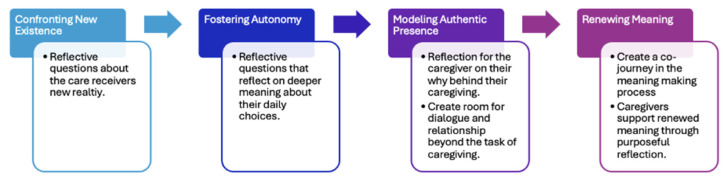
ETIC framework expanded.

To review, the ETIC framework includes four concepts: 1. Confronting new existence through the use of reflective questions, 2. fostering autonomy through the use of reflective and challenge questions, 3. modeling authentic presence through self-reflection, and 4. renewing meaning through mutual relationships; caregivers support renewed meaning through purposeful reflection with older adults about how to create new meaning in these circumstances (see Figure 2).

To utilize the ETIC framework, it is recommended that caregivers and skilled care communities engage in using Lieher and Smith’s [20] story theory or other narrative collection processes, which were developed in part to improve health outcomes and get to know the person. Capturing important biographical information on education, health history, family history, trauma and patient preferences helps caregivers and older adults build a human-centered care approach. The details that are captured in the life story should inform caregivers about potential trauma points in the person’s life course and how they may impact their attitudes, behaviors, and ambitions around engagement in their current health status [20]. Additionally, this trauma information gives understanding about the reasons behind certain behaviors and ways of being. To implement this practice, it is recommended that a life story is posted somewhere in the room or living space. This is important for new caregivers to have instant access and read it when entering the caregiving space, given the high turnover in these capacities. Caregivers that can quickly learn the background of the individual will be encouraged to integrate this knowledge with the care they offer. Training a caregiver to engage in the story through the ETIC framework overcomes any potential challenges of this model. A case study is provided here to show how the ETIC framework can be applied and how having a narrative or life story about an individual is useful and needed to apply this approach. While the case study presented is hypothetical in nature, the authors have personal experience working with family members and clients in practice who have experienced similar behaviors. The ETIC framework is intended to help improve care outcomes by acknowledging the existential experience of the individual.

The four components of the ETIC framework are applied to the case of Roger (Roger is not a real client, but rather a composite of a lot of different clients the authors have engaged with). Roger is a Vietnam veteran in his mid-seventies with a long history of diabetes and exposure to Agent Orange during the war. Because of his Agent Orange exposure, Roger receives a Veterans Administration disability pension. Roger’s early childhood was marked by significant physical and emotional abuse, leading him to leave home at age sixteen. At that time, he moved in with family friends. At nineteen years old, Roger was drafted into military service and, upon returning home, married. He rarely spoke about either his wartime or childhood trauma. Although Roger was known as someone who was friendly and helpful, he struggled to form deep relational connections and tended to focus primarily on day-to-day function rather than reflection or long-term meaning. In his seventies, Roger experienced a cardiac event that resulted in an extensive hospital stay. While in the hospital, Roger’s wife of 40+ years passed away. Understandably, this was a huge loss and a confusing turn of events for him. About six months after leaving the hospital, he suffered a stroke that left him with physical and speech disabilities. He was transferred to a skilled nursing facility for treatment and physical therapy; this marked a significant disruption to his sense of independence and identity. The stroke ultimately left him living permanently in the skilled nursing facility. The ETIC framework will be applied to Roger’s experience, and a detailed explanation will be provided on how to use it.

### 5.1. Confronting New Existence

The first component of the ETIC framework, confronting new existence, uses reflective questioning. For Roger, transitioning from independent living to a skilled nursing facility represents a profound existential and identity shift. A core aspect of how Roger saw himself was independent and self-reliant. These characteristics were also how Roger dealt with much of the trauma he experienced in early childhood and during the war. The move to a skilled nursing facility represented a huge shift for him. Although Roger recovered most of his speech following his cardiac event, periods of anxiety and worry were accompanied by a noticeable stutter.

Caregivers can support Roger by inviting reflection about the meaning of this life change rather than focusing exclusively on physical recovery. Reflective questions such as, “What has it been like for you to adjust here?”; “How does this stage of life feel different from what you expected?”; “What support do you need?”; or “What is one thing that helps you feel better?” are useful and create space for Roger to process his experience, thus reducing his anxiety. This type of questioning also lets a caregiver know if further services such as counseling might be helpful. Individuals entering skilled care or home care are confronted repeatedly with their medical limitations; yet caregivers rarely ask them to talk about the transition itself. Therefore, these reflective questions help Roger begin naming and integrating his new reality, which is foundational to the ETIC framework.

### 5.2. Fostering Autonomy

The second component, fostering autonomy, uses reflection and challenge combined. Given Roger’s history of trauma and limited relational engagement, supporting autonomy affirms his capacity for choice even within the constraints of his medical condition and care environment. While autonomy is a core principle in person-centered care of older people, it is often poorly understood in practice [21]. For older adults, admission to a skilled nursing facility or formal home care results in a felt sense of loss of autonomy, regardless of the quality of care provided.

In the case of Roger, his perception of his own autonomy was significantly compromised when he was transferred to skilled nursing care. The early experience of skilled nursing reinforced Roger’s feelings of dependence and loss of control. This triggered a trauma response from his past, given that independence and self-reliance were how he coped with his past traumas. Caregivers must recognize that care practices are about preserving the experience of autonomy and providing physical assistance. Roger may benefit from reflective questions that reestablish his sense of agency, such as, “What feels most important for you to have control over right now?” or gently challenge passive resignation by asking, “What would make today feel like a good day for you?” This line of questioning invites Roger to reflect and challenges him to articulate preferences, find a new direction for this experience, and participate more actively in shaping his daily experience of care, rather than feeling like care was just happening to him.

### 5.3. Modeling Authentic Presence

The third component of the model, modeling authentic presence, encourages caregivers to engage in ongoing self-reflection, particularly around their motivations for caregiving and their emotional responses to care relationships, so that they can be authentic in their work. Caregivers are asked to reflect on their “why’ behind their “what”. Why are they doing this position? Self-awareness supports greater emotional attunement with older adults This is especially important in the case of Roger. Roger did not transition well to skilled care. As previously stated, Roger had a history of avoiding emotional expression and relational depth; for a person like Roger, his only relational connection right now is the caregiver. Relationships are just as important to recovery as the physical aspects of care.

Beyond attending to physical needs, caregivers demonstrate authentic presence by acknowledging Roger’s identity and lived experience. For example, a caregiver might say, “I see that you are a Vietnam veteran; you seem really proud of your service.” If appropriate, the caregiver might facilitate an introduction with another veteran in the facility, fostering relational safety through shared experience. Caregivers can model authentic presence through limited, appropriate, and honest self-reflection, such as, “Some days I have a hard time adjusting to changes too. It helps me to take one step at a time.” These moments communicate shared humanity without placing emotional burden on Roger, thus building trust and relational depth.

### 5.4. Renewing Meaning Through Mutual Relationships

The final component of the ETIC framework, renewing meaning through mutual relationships, is about cultivating new relationships, even in a skilled care or home environment. For Roger, small intentional moments of attunement by caregivers can have a significant impact. If caregivers remain attentive to moments when Roger demonstrates adjustment to his situation, contribution, or engagement with others, they are encouraged to point that out.

For example, a caregiver might reflect, “You really made a difference in that person’s experience today. I noticed you took time to talk to him.” Such reflections reinforce Roger’s role as an active participant in relational life rather than solely a recipient of care. For home care recipients, depending upon their situation, they may have limited social interaction. In this case, the caregiver might acknowledge how the older adult impacts them personally. For example, in Roger’s case, caregivers can acknowledge how Roger’s actions positively affect them. When Roger begins dressing himself, a caregiver might say, “I appreciate that you got yourself dressed today. That really made the difference in our morning.” Through these mutual acknowledgments, Roger may start to connect with meaning, purpose, and relational identity even within his new skilled nursing environment.

## 6. Conclusions

Confronting new existence, finding autonomy in new circumstances, engaging in authentic presence, and establishing new meaning are all elements grounded in existential theory. When integrated with a trauma-informed perspective, these concepts challenge caregivers to move beyond task-focused care and engage more fully with older adults who have experienced trauma. Caregivers using the ETIC framework learn to relate and respond differently to the people they care for, thus supporting older adults whose sense of self, safety and meaning has been disrupted by trauma, illness and loss. The ETIC framework encourages caregivers to slow down, not just be physically present, but emotionally present. In practice, it encourages acknowledgement and reflection of fear, grief, confusion, and even anger, recognizing that older adults are experiencing a new existential reality. It encourages caregivers to understand difficult behaviors as trauma responses rather than rudeness or defiance. Also, caregivers focus on choices that individuals still have and encourage them to find agency within those choices still available to them.

Limitations and obstacles may become evident through the process of incorporating ETIC. As previously mentioned, there is high turnover among caregivers in healthcare, and the process of training may be short and inadequate for incorporating new frameworks of care like ETIC. However, posting a personal story or narrative that is easily accessible to view can help a caregiver develop a relationship quickly. When person-centered care incorporates this conversation starter or this understanding of a person’s life experience, it can aid in overcoming objections to recommended care options through an existential process, leading to improved care and compatibility. A second limitation is that the ETIC framework may not always be successful, given that mood, temperament, physical issues and limitations may still lend itself to an uncooperative situation. Older adults needing care may not always be willing to do the requested tasks required for their best interests. All care approaches have challenges under these circumstances that are not under the caregiver’s control. ETIC is a theoretically based conceptual framework offering a lens for guiding trauma-informed practice with older adults in caregiving environments. Future research exploring the efficacy of ETIC with an older adult population using both qualitative and quantitative methods would be valuable. Qualitative research provides lived experiences, acceptability, and contextual relevance, while quantitative methods provide outcome measurement and intervention effectiveness. Such work would not only strengthen the evidence base of ETIC, but also provide more research on trauma-informed care for older adults.

The ETIC framework reframes caregiving as a shared human encounter in which presence and reflection serve as essential skills. Caregivers engage in mutual presence. ETIC offers an approach to address both the psychological impact of trauma and the existential disruption experienced in the formal caregiving process. It is a bridge between trauma-informed care and existential understanding, offering a compassionate approach to working with older adults who have experienced profound life disruptions.

## Data Availability

No new data were created or analyzed in this study. Data sharing is not applicable to this article.
